# Decoding RNA–protein interactions using high-throughput methods

**DOI:** 10.1080/15476286.2026.2623240

**Published:** 2026-01-28

**Authors:** Marianne Régis, Paola Pulcina, Dmitry A. Kretov

**Affiliations:** a Department of Molecular Biology, Medical Biochemistry and Pathology, Faculty of Medicine, Université Laval, Québec, QC, Canada; b Oncology Division, CHU de Québec-Université Laval Research Center, Québec, QC, Canada; c Université Laval Cancer Research Center, Québec, QC, Canada

**Keywords:** RNA–protein interactions, RNA-binding proteins, RBPs, microRNAs, high-throughput approaches, massively parallel binding assays, massively parallel reporter assays, sequence space

## Abstract

RNA-binding proteins (RBPs) constitute a diverse class of proteins essential for every stage of the gene expression process. Many RBPs are also linked to human diseases and pathologies. Understanding the molecular grammar of RNA–protein interactions is critical for deciphering the regulatory RNA code. This review provides a comprehensive overview of Massively Parallel Binding Assays (MPBAs), high-throughput techniques that use large libraries of RNA or protein variants to systematically investigate RNA–protein interactions. We describe the underlying principles of both *in vitro* and *in vivo* approaches, their applications, as well as their strengths and weaknesses. We conclude by outlining future directions and challenges in the field that will help drive the development of novel methods to better understand the RBP recognition code.

## Introduction

As soon as nascent mRNA emerges during transcription, it is bound by diverse RNA-binding proteins (RBPs) that regulate its processing, modification, stability, translation, and localization [1,2]. These RBPs act in concert with each other on mRNA, assembling into dynamic messenger ribonucleoprotein (mRNP) complexes that are continually remodelled throughout the mRNA lifecycle [3,4]. Over the past decade, significant progress has been made in identifying the full repertoire of RBPs expressed across different cell types and organisms. In humans, RBPs constitute one of the most highly expressed protein classes, comprising more than 1500 distinct members [5]. These proteins play essential roles in shaping gene expression during development and maintaining cellular homoeostasis. Moreover, RBP functions are often dysregulated in diseases, including cancer and neurodegenerative disorders [6–8].

RBPs can bind to mRNA either through direct RNA–protein interactions or via small RNAs [9]. The latter mechanism could be exemplified by microRNAs, which guide Argonaute proteins to their mRNA targets, and small nuclear RNAs (snRNAs), which confer sequence specificity to the spliceosome [10,11]. Small RNA-guided RNA recognition is relatively well understood, largely because of the more predictable nature of RNA–RNA base pairing [12]. In contrast, direct RNA–protein interactions are more complex and challenging to predict [13,14]. RBPs recognize their RNA targets through a variety of RNA-binding domains (RBDs), such as canonical RRM (RNA Recognition Motif), KH-domain (K-Homology domain) and CSD (Cold Shock Domain). Importantly, individual RBDs often bind weakly on their own, but most RBPs have a modular architecture with an average of 2–4 RBDs, which together increase binding specificity and affinity [15–17]. Additionally, RBDs often function in combination with auxiliary domains that modulate their binding to RNA. For instance, intrinsically disordered regions (IDRs) are prevalent in RBPs and play critical roles in RNA binding [18–21]. Finally, several metabolic enzymes and transcription factors (TFs), traditionally not thought to bind RNA, have been shown to bind mRNAs directly [22,23]. Many such experimentally discovered RBPs lack any canonical RBDs, highlighting the limitations of domain-based annotations and revealing that RNA-binding capacity is more widespread and functionally diverse than previously appreciated [24,25].

In parallel, significant progress has been made in deciphering the *cis*-regulatory RNA code that governs mRNA stability, translational efficiency, splicing, and localization [26–32]. However, the functionality of this code depends on recognition by *trans*-acting factors like RBPs and small RNAs. Importantly, the same *cis*-element may be interpreted differently depending on cellular context [33,34]. Although most RBPs exhibit relatively low tissue specificity, their function can vary significantly with differences in expression levels, post-translational modifications, and alternative splicing [35–39]. Moreover, RBPs regulate mRNAs in a combinatorial manner, so even in cells with similar RBP pools, mRNAs with different 3′UTRs can be regulated differently depending on the specific set of RBPs bound to them [40]. Therefore, to fully understand the contribution of an entire 5′ or 3′UTR of a particular mRNA to its function, it is important to determine how each *cis*-element is recognized by *trans*-acting factors, and how their interplay is regulated within specific cellular contexts. This knowledge would enable predictive modelling of mRNA behaviour and guide the design of programmable RNA molecules for the needs of biotechnology and RNA therapeutics [41].

Among the methods used to identify RNA-binding sequences of RBPs, crosslinking and immunoprecipitation (CLIP)-based approaches are the most widely used [42,43]. A major advantage of CLIP is its ability to capture endogenous RNA–protein interactions. However, CLIP is not well suited for obtaining precise quantitative information such as binding affinities (*K*_D_ values), or for distinguishing between stable and transient interactions. To date, only one CLIP-based method, KIN-CLIP, has been developed to profile the kinetics of RNA–protein interactions in living cells. However, it is technically challenging and has so far been applied only to one protein, DAZL (Deleted in Azoospermia Like) [44]. Moreover, CLIP analyses tend to prioritize strong, high-affinity interactions while overlooking low-affinity interactions that may still be functionally relevant. Similar to TFs, which bind their DNA targets across a continuum of affinities [45–47] rather than in an all-or-none fashion, RBPs likely recognize their RNA targets through a spectrum of affinities shaped by both RNA sequence and structure. As demonstrated for TFs, low-affinity interactions are often evolutionarily selected, and deviations from these optima can result in developmental defects [48,49]. Similarly, the RBP C5, the protein subunit of RNase P, an enzyme responsible for processing precursor tRNAs, binds its physiological RNA targets with affinities that fall in the middle of its overall binding spectrum, highlighting their biological relevance [50]. This observation suggests that other RBPs may also rely on a broad range of binding affinities to carry out their functions effectively.

Here, we define MPBAs (Massively Parallel Binding Assays) as high-throughput methods that use large libraries of RNA or protein sequence variants to investigate RNA–protein interactions. While these approaches rely on artificial systems and do not directly probe endogenous RNA–protein interactions, they enable exploration of a much broader sequence space than is possible with endogenous transcripts, which are inherently limited in diversity. Consequently, comprehensive characterization of RBP specificities and binding affinities requires the use of larger, more complex RNA libraries. MPBAs are distinct from MPRAs (Massively Parallel Reporter Assays), which primarily measure the effects of *cis*-elements on RNA function (e.g. stability, splicing, localization, or translation) and do not directly assess the association of specific *trans*-factors with RNA [28]. The use of synthetic RNAs in MPBAs also enables the study of low-abundance transcripts that are difficult to analyse endogenously. Furthermore, because MPBAs can use RNA sequences derived from any class of transcript, including mRNAs, lncRNAs, and other noncoding RNAs, they offer a general framework for quantifying how RBPs interact with diverse RNA substrates. In this review, we describe MPBAs that use synthetic libraries of RNA or protein sequence variants to characterize RNA-protein binding. We highlight approaches that explore large sequence spaces, quantify binding determinants at high resolution, and provide kinetic measurements of RNA–protein interactions. We begin by describing *in vitro* MPBAs that quantify RBP-RNA binding interaction and dissect sequence binding determinants at high resolution. We then discuss recent advances in the quantitative *in vivo* analysis of RNA–protein interactions. Finally, we highlight the main challenges, outline outstanding questions in the field, and speculate on future directions to address them. We will not discuss CLIP- or RNA-editing-based methods for analysing RBP binding to endogenous RNA, as they do not provide quantitative information about the kinetics of RBP binding and have been extensively reviewed previously [42,43,51].

## General considerations about the design of MPBAs

In principle, any interaction between an RBP and RNA can be characterized by three key parameters: binding specificity, affinity, and interaction kinetics [52,53]. To fully describe the properties of an RBP, it is essential to measure all these parameters, ideally within the natural cellular contexts in which they occur. While comprehensive characterization of a single RBP is possible using a range of specialized low-throughput techniques, achieving this at high throughput has only become feasible with advances in DNA synthesis and sequencing technologies. These innovations have enabled the generation of complex sequence libraries and the detailed analysis of enriched sequence pools. Generally, all MPBAs for studying RBP binding specificity involve three main steps: (1) generation of a library of RNA motifs and the RBP of interest (or a library of RBPs), (2) incubation of the RNA library with the RBP, and (3) identification and analysis of the RNA sequences bound by the protein. Some MPBA methods are better suited for probing RNA sequence variation, others for exploring protein variants, and some allow simultaneous interrogation of both components. Existing techniques differ in the specific strategies used to perform each of these steps.

The design of the RNA library is a critical component as it defines the sequence space available for RBP binding. Most individual RBDs recognize relatively short motifs, typically 4 to 8 nucleotides in length, making it feasible to use fully randomized libraries that encompass all possible variants within this range. However, full-length RBPs, which are often composed of multiple domains, can interact with longer RNA sequences (20–40 nucleotides) [54], where binding motifs may occur adjacently, be separated by spacer regions, or be embedded within specific secondary structures. The use of fully randomized 40-nucleotide libraries poses a significant challenge, as the theoretical sequence space (~10^24^ molecules) is astronomically large and cannot be fully represented during synthesis or comprehensively sampled by sequencing. As a result, alternative strategies are often used, such as shorter sequences, partially mutagenized libraries, or fragments of endogenous transcripts, to reduce sequence complexity while maintaining high sequence diversity necessary to characterize RBP binding preferences. As Next-Generation Sequencing (NGS) is the most commonly used readout in MPBAs, sequencing depth becomes an important consideration. Achieving approximately 100–1000 reads per variant is generally desirable to obtain reliable enrichment estimates. Therefore, depending on the complexity of the library, sequencing depth should be adjusted to ensure sufficient and uniform representation of all variants.

The incubation step also varies across MPBA techniques. It may occur *in vitro* or *in vivo*, and factors such as incubation time and the concentrations of RNA and protein introduce additional layers of variation. Finally, these methods differ in how they enrich RNA molecules that interact with RBPs versus those that do not and in the type of readout used. Readouts may include sequence enrichment, survival selection, reporter protein expression, or RNA editing/modification. We have summarized these three major steps of all methods discussed in this review Table 1.

**Table 1.** Comparison of different *in vitro* and *in vivo* MPBAs.

	*Method*	*RNA*		*Protein*	*Incubation features*	*Analysis*
*IN VITRO*	SELEX	Randomized 20–100 nt library		Purified recombinant GST-tagged, His-tagged, or untaggedprotein (the latter immobilized using specific antibodies)	~4–8 rounds of selection	Sanger
	SEQRS	Randomized 20-nt library			5 rounds of selection sequences analysed every cycle	NGS
	HTR-SELEX	Randomized 40-nt library			4 rounds of selection	NGS
	Phage Display (PD)	Immobilized RNA of interest		Library of protein variants (up to 10^8^ fused to phage coat protein	2–4 rounds of selection	Sanger
	PD-SELEX	Randomized 20-nt library			6 rounds of selection Library against library selection	NGS
	RNAcompete	~240,000 unique (30–41 nt) RNAs		Purified recombinant GST- and/or SBP-tagged protein	Single incubation step Excess of RNA	Microarray
	RNAcompete-S	Randomized 40-nt library				NGS
	RNA Bind-n-Seq	Randomized 40-nt library			Range of protein concentrations	NGS, 15–20 million reads/library
	nsRBNS	Targeted library of ~110–120-nt fragments of endogenous RNAs				NGS
	Endo-RBNS	Randomized 8- or 14-nt library		Pulldown of endogenous or FLAG–HA– tagged protein from mammalian cells or tissues	2-rounds of selection Range of protein concentrations	NGS, 30,000 reads/sample
	HiTS-Kin	Randomized up to 8-nt library		Purified recombinant protein	Time-resolved kinetics Separation of complexes by PAGE	NGS, >100 reads per variant
	HiTS-Eq				Range of protein concentrations Separation of complexes by PAGE	
	RNA-MaP	Library of 1,000,000 variants		Purified recombinant and fluorescently labelled protein (with Cy3, mOrange, etc).	Biotin-streptavidin roadblock Range of protein concentrations	Fluorescence on the flow cell (Illumina)
	HiTS-RAP	Library of 1000–10,000 variants			Tus-TER roadblock Range of protein concentrations	Fluorescence on the flow cell (Illumina)
	RNA-CHAMP	Library of 10,000 variants			Tus-TER roadblock Range of protein concentrations	Fluorescence on the flow cell (conventional microscopy)
	RNA-MITOMI	Library of 100 variants			Reaction in custom MITOMI device Range of protein concentrations	Fluorescent microarray scanner
*IN VIVO*	Translation initiation block	Library of RNA variants (~11) in reporter mRNA	Protein variants ectopically expressed in *E. Coli*		*E. Coli* RNA library before Shine-Dalgarno Negative readout of RNA–protein interactions	*β*-galactosidase activity, Sanger
	Transcriptional antitermination	Tested RNA is positioned before terminators	Protein of interest is fused to λN antiterminator protein		*E. Coli* Positive readout of RNA–protein interactions	*β*-galactosidase activity, Sanger
	Tat-hybrid assay	RNA of interest is positioned before GFP	Library of protein variants fused to Tat protein		Mammalian cells ~3 rounds of selection	Fluorescence, Sanger
	Yeast three-hybrid	Library of 50–150 nt RNA fragments	Protein fused to transcription activator domain		Yeast MCP-DNA-binding domain is required Binding at the promoter regions	*β*-galactosidase activity, Sanger, NGS
	MPRNA-RIP	Library of designed 110–140-nt RNA fragments	Endogenous RBP or ectopically expressed protein with FLAG or HA-tag		Mammalian cells Enrichment via IP under native conditions RNA library is in the 3’UTR	NGS
	MPRNA-IP	Tiled 157-nt fragments of RNAs of interest			Mammalian cells Enrichment via IP Formaldehyde crosslinking	NGS
	RBPscan	Randomized or designed 7–8-nt library	Protein fused to the catalytic domain of ADAR		Mammalian cells, zebrafish, yeast RNA library is in the 3’UTR Readout via RNA editing	NGS

## *In vitr*o MPBAs

### Systematic evolution of ligands by exponential enrichment (SELEX)

**Figure 1.**
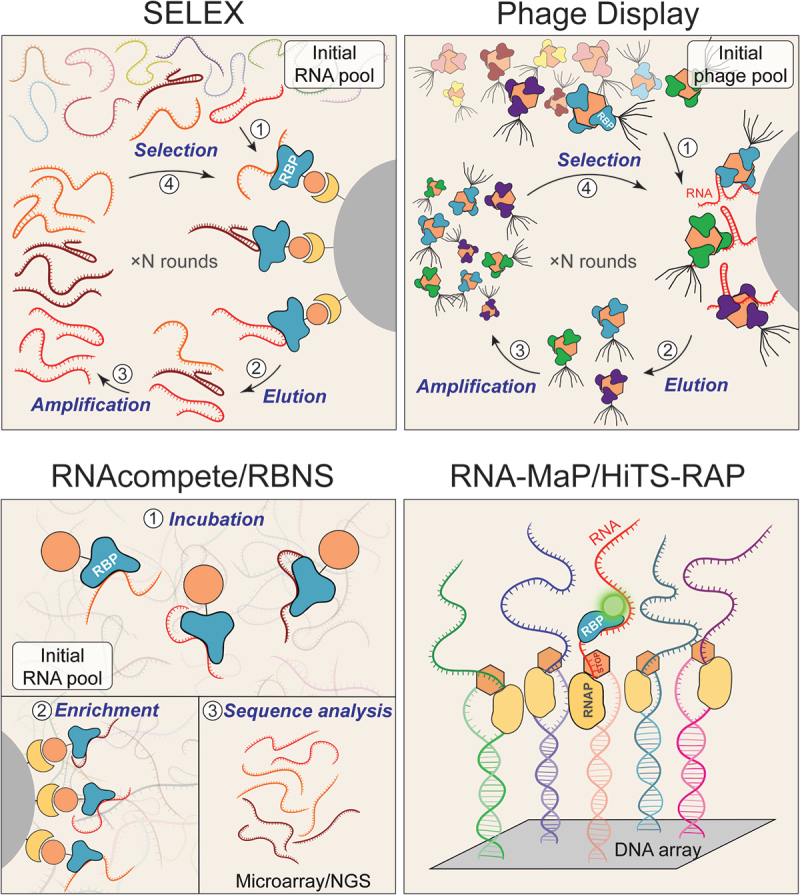
Schematic representation of *in vitro* MPBAs.

Systematic Evolution of Ligands by Exponential Enrichment (SELEX), developed in 1990 by the groups of Larry Gold and Jack Szostak, was the first high-throughput method used to identify RNA ligands for T4 DNA polymerase and RNA aptamers that bind small molecules [55,56]. It was later adapted to identify preferred binding motifs of canonical RBPs [57–61]. The SELEX process begins with the synthesis of a diverse pool of DNA molecules (10^13^-10^14^) containing randomized regions (~20–100 nucleotides) flanked by constant sequences required for PCR amplification. This DNA pool is then *in vitro* transcribed into RNA and incubated with an RBP of interest [55,62] (Figure 1(A)). RNA molecules that bind the protein are isolated using affinity chromatography, nitrocellulose filter-binding assays, or paramagnetic beads, and then reverse-transcribed, PCR-amplified, and *in vitro* transcribed back into RNA for the next round of selection. Through iterative cycles (typically 4–8), the sequence diversity in the library is gradually reduced until a small number of high-affinity RNA molecules that bind to the RBP are enriched. The final ‘winner’ sequences are analysed using conventional sequencing. Due to this limitation, SELEX-based methods are biased towards the highest-affinity ‘winner’ motifs and are not quantitative, preventing direct comparisons of RBP binding to many RNA sequences in parallel. As a result, motifs with lower binding affinity are often missed.

A) SELEX. A pool of *in vitro* synthesized RNAs is incubated with the RBP of interest. RBP–RNA complexes are isolated by affinity pull-down, and bound RNAs are eluted, reverse-transcribed, and PCR-amplified. The enriched RNA pool is regenerated and subjected to iterative selection rounds until only high-affinity RNA sequences remain.

B) Phage display. A pool of phages displaying different RBPs on their surface is incubated with the RNA of interest. RBP–RNA complexes are pulled down, and bound phages are amplified by infection of *E. coli*. Several iterative rounds are performed until only a few high-affinity clones are enriched.

C) RNAcompete and RBNS. A pool of RNA molecules is incubated with RBPs at different concentrations (RBNS) or with an excess of RNA (RNAcompete). RBP–RNA complexes are purified via affinity tags, and the bound RNA sequences are analysed either by microarrays (RNAcompete) or by NGS (RBNS and RNAcompete-S).

D) RNA-MaP and HITS-RAP. In these approaches, a defined RNA pool is *in* *situ–*transcribed from a custom DNA array immobilized directly on the NGS flowcell. Each RNA molecule remains tethered to its template DNA via a terminal roadblock. Fluorescently labelled RBPs are incubated with the flow cell, and binding is visualized and quantified by fluorescence microscopy.

To address this limitation, SEQRS (*in vitro* selection, high-throughput sequencing of RNA, and sequence specificity landscapes (SSLs)) and HTR-SELEX (High-Throughput RNA-SELEX) have been developed [63,64]. SEQRS and HTR-SELEX are conceptually similar to SELEX but use NGS for the analysis of enriched sequences. These approaches use fewer selection rounds (~3–5), which helps preserve more diverse sequence pools and provides more detailed information about binding preferences. Using randomized 20-mer sequences, SEQRS has been successfully applied to identify binding motifs of several members of the PUF (Pumilio and FBF)) protein family. SEQRS has also been used to assess cooperative interactions between two RBPs, such as the effect of CPB-1 (Cytoplasmic Polyadenylation Element Binding protein) on FBF-2 (Fem-3 mRNA binding protein 2), which are both involved in the control of stem cells and sex determination in *C. elegans* as well as between two translational repressors Nanos and Pumilio, in *D. melanogaster* [63,65]. HTR-SELEX has been applied to identify binding motifs of 86 RBPs using randomized 40-mer sequences and has identified not only short linear motifs, but also structural motifs and gapped motifs [64].

In summary, although SELEX-based approaches use large RNA pools and sequence diversity, which allow for the unbiased selection of RNA sequences capable of adopting specific conformations, identification of high-affinity RBP-binding motifs, and potential discovery of alternative conformations with similar binding, they also have some limitations. Specifically, with the exception of SEQRS, these techniques do not provide information on sub-optimal binding sequences and none of them infrorm about the kinetics of RBP–RNA interactions. However, this limitation can potentially be addressed by analysing earlier rounds of selection, which may help to identify sub-optimal binding motifs that are lost in later rounds.

### Phage display (PD)

Phage Display (PD) is a tool used to select protein variants based on their ability to bind a target substrate. In this technique, the protein of interest is genetically fused to a bacteriophage coat protein, allowing it to be displayed on the surface of the phage [66]. This enables the presentation of large libraries of protein variants, which can be screened for their binding to a target of interest (a protein, DNA, or RNA). Bound phages are then isolated and amplified by infecting *E. coli*, allowing for iterative rounds of selection and enrichment [66] (Figure 1(B)). PD is well suited for elucidating the contribution of individual amino acids within RBPs towards RNA recognition. PD has been shown to be effective for selecting functional RBPs from large libraries of variants, including those based on the RRM domain of spliceosome component U1A [67]. In addition, PD has been successfully applied to identify and clone RBPs from cDNA libraries [68].

A recently developed method, PD-SELEX, integrates PD with SELEX to enable simultaneous screening of RBP and RNA variants [69]. Libraries of phage-displayed RBP variants and synthetic RNAs containing a universal sequence are first generated. After incubating the two libraries together, selection is performed through two sequential affinity purification steps: first isolating phages displaying RBPs, then capturing RNA–protein complexes via the universal sequence present in all RNA variants. The enriched RNAs are reverse-transcribed, PCR-amplified, and *in vitro* transcribed again, while the enriched phages are amplified in *E. coli* prior to the next selection round. The PD-SELEX approach has been used to identify novel orthogonal interaction pairs between L7Ae variants and the K-turn RNA motif [69,70]. This demonstrates that PD-SELEX can be applied for the discovery and optimization of RBP–RNA pairs. However, the number of RBP variants that can be displayed on the phage may present a limiting factor. As the library size used in phage display is limited by the transformation capacity of *E. coli* (10^9^-10^10^), alternative approaches for testing large protein libraries (up 10^13^) entirely *in vitro* have been developed, including ribosome display [71] and mRNA display [72]. For instance, mRNA display, which relies on the formation of a covalent bond between a synthesized protein and its encoding mRNA via puromycin, has been used to identify novel peptides that bind to the boxB hairpin [73].

### RNAcompete

RNAcompete is a high-throughput *in vitro* method to study the binding preferences of RBPs across many RNA sequences in parallel [74,75]. The classical protocol involves generating a non-random RNA pool composed of two independent sets of sequences (~30–40 nucleotides in length), each containing all possible 9-mer sequence variants, enabling independent assessment of how sequence context influences RBP binding. DNA oligonucleotides encoding this RNA pool are synthesized using Agilent 244K microarray technology and subsequently *in vitro* transcribed. The RNA pool is then incubated with a purified recombinant GST-tagged RBP of interest, followed by a single-step pulldown of RNA–protein complexes (Figure 1(C)). Importantly, binding is performed in the presence of a ~ 75-fold molar excess of RNA relative to the protein to ensure that multiple RNA sequences compete for the same RBP. This competition allows RNA molecules to exchange based on their *K*_OFF_-rates, assuming uniform *K*_ON_-rates across sequences in the absence of competition from other factors. However, this titration regime is not optimal for determining the dissociation constant (*K*_D_) or accurately measuring RBP–RNA association kinetics [53]. The RNA recovered from the pulldown is fluorescently labelled with Cy5 and hybridized together with a Cy3-labelled input RNA pool onto Agilent 244K microarray matching the original design, which enables quantification of the relative enrichment of RNA in the pulldown fraction compared to the input [75]. The output is subjected to Z-transformation to normalize probe intensities and then analysed using *k*-mer enrichment (typically 7-mers) to identify sequence motifs present in top binding sequences. In a large-scale study, RNAcompete was applied to characterize the binding preferences of 205 canonical RBPs from various species [76]. More recently, it has been used to investigate the binding properties of 492 unconventional RBPs lacking canonical RBDs [77]. Additionally, RNAcompete data have been leveraged to generate a comprehensive resource of binding motifs for thousands of different eukaryotic RBPs containing RRM and KH domains based on the homology of their RNA-binding regions [78]. These motifs are aggregated in the CisBP-RNA database, which compiles experimentally determined and predicted RBP binding motifs across eukaryotes [79]. Despite the advantages of RNAcompete, such as its relatively low cost and a straightforward experimental procedure, the use of microarrays for RNA synthesis and analysis limits the number of probes that can be evaluated. Additionally, the RNA pool used in RNAcompete provides limited representation of secondary structures and is biased towards unstructured motifs. This limitation has been overcome by the development of RNAcompete-S in which the RNA pool is synthesized using a 40-nt randomized template and analysed using high-throughput sequencing [80]. Another advantage of RNAcompete-S is the absence of flanking priming sequences commonly used in complex RNA pools to facilitate library preparation for NGS. These sequences can impact secondary structure formation or be directly recognized by RBPs. Therefore, while RNAcompete-S minimizes this bias in RBP binding, this feature comes at the cost of a more laborious library preparation procedure.

### RNA bind-n-Seq (RBNS)

RNA Bind-n-Seq (RBNS) is a quantitative, biochemical method for assessing RNA–protein interactions, adapted from the original Bind-n-Seq technique developed for DNA-binding proteins [81,82]. In the typical RBNS protocol, a pool of short synthetic RNAs is generated by *in vitro* transcription (Figure 1(C)). This pool contains a fully randomized region (20 to 40 nt) flanked by constant Illumina adaptor sequences needed for high-throughput sequencing. The use of a fully randomized region enables a comprehensive assessment of RNA sequence space, capturing both short linear motifs and potential secondary structures that can form in *cis* within RNAs of this length. Despite the advantages of using longer RNA sequences (40 nt) for capturing the effects of RNA secondary structure, most RBNS experiments are performed with shorter sequences, typically around 20 nt or shorter, as they allow more efficient identification of short linear binding motifs [21,83,84]. Additionally, a variant of RBNS known as natural sequence RBNS (nsRBNS) has been developed that utilizes natural sequences from the endogenous transcriptome [85–87]. This approach enables the analysis of RBP binding to longer RNA fragments (~100 nt) while maintaining relatively low library complexity, ensuring full representation of all sequences. A key advantage of RBNS over other methods, such as RNAcompete, is its use of multiple RBP concentrations, allowing detection of interactions across a broad affinity range, from low nanomolar to high micromolar. At lower protein concentrations, high-affinity binding sites are preferentially bound, while higher RBP concentrations saturate these sites, revealing sub-optimal binding sites [21,86,88]. The RNA pool is incubated with a recombinant RBP purified from *E. coli* until binding equilibrium is reached. This concentration-dependent binding behaviour enables estimation of dissociation constants (*K*_D_) for specific sequence elements. RBP–RNA complexes can be isolated using methods such as pull-down with specific antibodies (if the RBP is tagged), electrophoretic mobility shift assay (EMSA), or double-filter binding assay. Bound RNAs are then purified, sequenced, and compared to the input library. In classical RBNS, binding preferences are quantified by calculating an enrichment score (‘R’ score) for each *k*-mer in the bound versus input fractions, resulting in a ranked list of preferred binding motifs. RBNS datasets generated by the ENCODE consortium are publicly accessible through the ENCODE Data Portal [84]. RBNS has been used to determine relative dissociation constants for the microRNA-induced silencing complex (miRISC) [89], and more recently, the method has been optimized to obtain absolute *K*_D_ values, including association and dissociation (*K*_ON_ and *K*_OFF_ rates) rates for Argonaute 1 (Ago1), a key component of the miRISC [88,90]. Recently, RBNS has been further extended to allow the use of RBPs immunoprecipitated from mammalian cells (endo-bind-n-seq), enabling the analysis of full-length proteins and potentially accounting for post-translational modifications [91].

### High-throughput sequencing kinetics (HiTS-Kin) and equilibrium (HiTS-Eq) approaches

High-Throughput Sequencing Kinetics (HiTS-Kin) is a biochemical approach developed to measure the kinetics of RNA–protein interactions and RNA processing across thousands of RNA sequence variants simultaneously [50,92,93]. Unlike methods such as RNAcompete or RBNS, which assess binding under equilibrium conditions, HiTS-Kin incorporates a time-resolved component to capture reaction kinetics. Another key feature of HiTS-Kin is that it measures the interaction of an RBP with all possible sequence variants within a randomized RNA pool. This enables experimental mapping of the complete RBP affinity landscape. However, to ensure sufficient coverage and sequencing depth, the length of the randomized region is typically limited to 6–8 nucleotides. In this approach, an RNA pool containing a fully randomized region within the RBP binding site is incubated with the RBP of interest, and samples are collected at multiple time points. The setup resembles traditional biochemical assays, such as EMSA or cleavage assays where reaction products are visualized by Polyacrylamide Gel Electrophoresis (PAGE), but is performed simultaneously for thousands of sequence variants of a specific RNA. By measuring the abundance of each variant in the unbound or unprocessed fraction over time, the method captures the relative reaction rates for each sequence. HiTS-Kin was originally applied to C5 to measure the kinetics of its association with all possible sequence variants at its binding site. This study revealed that the physiological targets of C5 do not correspond to its highest-affinity binding motifs but rather fall near the median of the overall affinity distribution [50]. A variant of HiTS-Kin, called HiTS-Eq (High-Throughput Sequencing Equilibrium), was later developed to measure kinetics under equilibrium conditions. Similar to RBNS, it uses varying RBP concentrations to assess binding preferences across different affinity ranges [94]. Recently, HiTS-Eq was used to demonstrate the bimodal binding behaviour of Rbfox2 to different RNA substrates, revealing two distinct protein conformations [95], and helped explain the extraordinary specificity of Rbfox2. Since the sequence space in HiTS-Kin and HiTS-Eq is limited to 8-mers (10^6^) and requires flanking constant sequences, these techniques are best suited for comprehensively characterizing known binding or cleavage sites in specific RNAs, rather than for discovering previously uncharacterized binding specificities.

### RNA on a massively parallel array (RNA-MaP) and high-throughput sequencing and RNA affinity probing (HiTS-RAP)

Quantitative analysis of RNA on a Massively Parallel array (RNA-MaP) and High-Throughput Sequencing and RNA Affinity Probing (HiTS-RAP) are two similar methods that were developed to enable accurate quantification of RNA-protein binding kinetics *in vitro* [96,97]. In these approaches, interactions between an RBP in solution and RNA immobilized on the surface of a NGS flow cell are detected using a fluorescent readout (Figure 1(D)). First, a DNA library encoding variable RNA regions, along with T7 promoter sequences, is attached to the surface of the flow cell and then amplified, generating clusters of unique sequences (~10^3^ molecules per cluster) that are identified by sequencing. This DNA library is then used for *in vitro* transcription to produce the corresponding RNA pool directly on the chip. Importantly, a transcriptional roadblock is incorporated at the end of each DNA template to stall RNA polymerase, tethering the newly synthesized RNA to its DNA template and preventing dissociation. In HiTS-RAP, the replication terminator protein Tus is used as the transcriptional roadblock, while RNA-MaP employs biotin-streptavidin complexes for the same purpose. The RBP of interest is fluorescently labelled and injected into the flow cell. After equilibrium is reached, fluorescence is measured at each location on the flow cell, where an increase in signal indicates stronger binding. Using the RNA-MaP approach, binding constants and dissociation kinetics of the MS2-binding protein have been measured for more than 10^7^ variants of the MS2 hairpin [96]. In HiTS-RAP, dissociation constants have been measured for thousands of RNA aptamers binding to green fluorescent protein (GFP) and the negative elongation factor E (NELF-E) [97]. The RNA-MaP approach has also been employed to measure the binding kinetics of Pumilio homology domains to a comprehensive library of its RNA binding motifs, enabling the development of an accurate thermodynamic model describing Pumilio-RNA interactions [98,99]. Additionally, RNA-MaP was used to determine the thermodynamic specificity of microRNA-target interactions [100]. To account for the natural context of assayed RNA sequences, RNA-MaP was also used to examine the binding preferences of Vts1, a member of the Smaug protein family, using a Transcribed Genome Array (TGA) composed of 100-nt fragments spanning the entire *S. cerevisiae* genome immobilized on the flow cell [101]. As highlighted by the examples above, RNA-on-chip assays are powerful tools that enable precise biochemical measurements across RNA sequence space [102,103]. However, they require sophisticated equipment that is not accessible to most laboratories. To overcome this obstacle, a similar approach, RNA-CHAMP (Chip-Hybridized Association-Mapping Platform), was recently developed. It is compatible with Illumina flow cells as well as conventional microscopes and has been successfully used to determine the binding properties of RNA-guided RNA-endonuclease Cas13d [104]. Finally, it should also be considered that many proteins possess dual RNA- and DNA-binding properties [105]. Therefore, the presence of DNA as a template and bridge to tether RNA on the chip can potentially affect the determination of binding constants. This can be overcome by using specialized microfluidic devices such as RNA-MITOMI (Mechanically Induced Trapping of Molecular Interactions), where *in vitro* transcription and binding assays take place in two separate chambers, as was demonstrated for the histone stem-loop binding protein [106]. Despite its advantages, the use of this approach is limited by its high cost and the need to fabricate customized MITOMI devices.

## *In vivo* MPBAs

### *Pioneering* in vivo *strategies for RNA–Protein interaction discovery*

**Figure 2.**
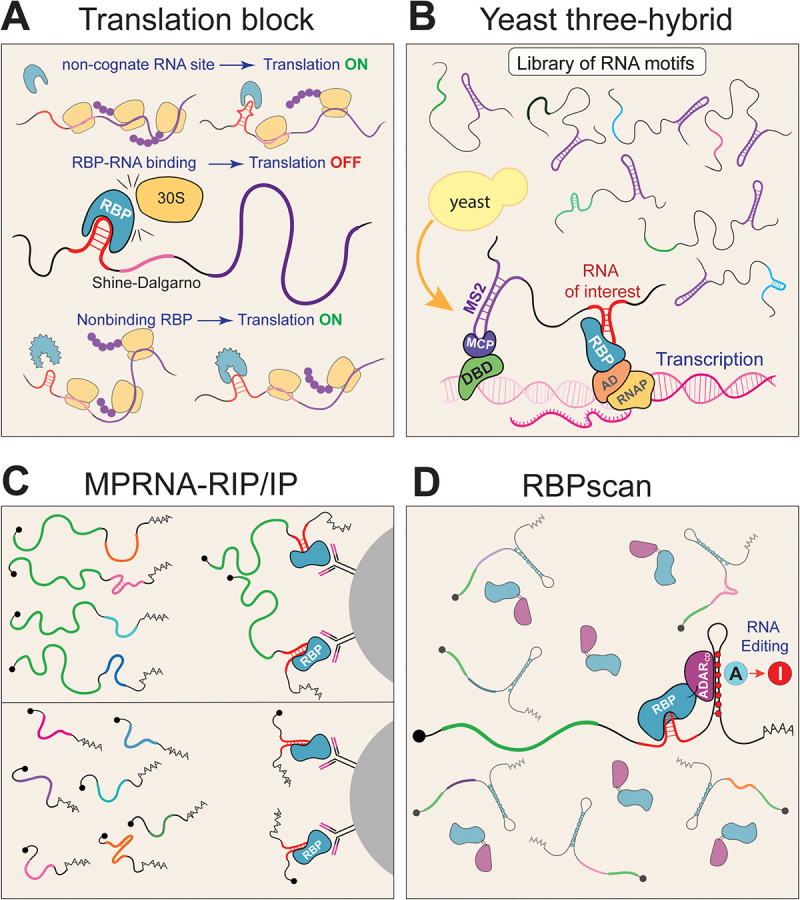
Schematic representation of *in vivo* MPBAs.

One of the first approaches developed to profile RNA–protein interactions *in vivo* was implemented in bacteria. These systems rely on either inhibition or activation of the synthesis of a quantifiable reporter protein, such as *β*-galactosidase, to measure RNA–protein binding. The first approach, translation initiation block, exploits the requirement for base-pairing between the Shine-Dalgarno (SD) sequence and the ribosome for efficient translation [107] (Figure 2(A)). Inserting the RNA sequence of interest upstream of the SD site allows bound proteins to sterically hinder ribosome access, reducing *β*-galactosidase expression. Originally, this approach was used to identify mutations in the HIV-1 Rev protein that affect binding to the Rev response element (RRE) stem-loop, as well as to study interactions involving the spliceosomal protein U1A [107], and it was later optimized to include a fluorescent readout [108]. Another class of approaches relies on RNA–protein-dependent transcriptional antitermination and has also been used to isolate peptides that specifically bind the HIV Rev response element [109]. Despite their original design and utility, these approaches are limited to bacteria due to fundamental differences in gene expression control between prokaryotes and eukaryotes (e.g. the lack of a Shine–Dalgarno sequence requirement in eukaryotes). One of the first *in vivo* approaches used in mammalian cells is the Tat-hybrid assay, which is based on the HIV Tat-TAR system [110–112]. In this method, a library of RBPs fused to the Tat protein is expressed, and the TAR (Tat response element) is replaced with the RNA sequence of interest. If an RBP-Tat fusion binds to the tested RNA, it recruits transcriptional machinery and activates the expression of a reporter gene, such as GFP . This approach has been applied to study several RNA–protein interactions, including Rev-RRE and SF1-U2AF65-branchpoint interactions [111,113].

A) Translational roadblock assay. The tested RNA motif is inserted near the Shine–Dalgarno sequence. When an RBP binds to this motif, it interferes with ribosome recruitment, leading to decreased translation of the reporter protein.

B) Yeast three-hybrid screen. Three components are co-expressed in yeast cells: a DNA-binding domain fused to MCP, an RNA of interest fused to MS2 hairpins, and the RBP of interest fused to a transcriptional activation domain. If the tested RNA and RBP interact, the transcription of a reporter gene is activated.

C) A library of RNAs, either carrying a reporter gene (MPRNA-RIP; upper panel) or expressed without a reporter (MPRNA-IP; lower panel), and containing variable test sequences, is introduced into cells. RNAs associated with the RBP of interest are then isolated by immunoprecipitation using specific antibodies. The recovered RNA fragments are quantified by NGS.

D) RBPscan. A library of mRNA reporters is expressed together with a fusion of the RBP and ADAR_CD_. When the RBP binds its target sequence, ADAR mediates A-to-I editing within the reporter, which is subsequently detected by sequencing as A-to-G conversations.

### Three-hybrid systems

The Yeast Three-Hybrid (Y3H) system is a genetic tool derived from the yeast two-hybrid assay, adapted to detect RNA–protein interactions *in vivo* [114]. It relies on the formation of specific RBP-RNA interactions to trigger transcriptional activation of a reporter gene, thereby enabling a rapid phenotypic readout of tested molecular interactions (Figure 2(B)). The system involves the expression of three components: (1) a hybrid protein composed of a DNA-binding domain (such as LexA) fused to the MS2 coat protein (MCP), which localizes to the promoter region of a reporter gene; (2) an RBP of interest fused to a transcriptional activation domain (such as Gal4); and (3) an RNA bait molecule containing both the conserved MS2 hairpin and variable RNA sequence of interest. When all components are expressed in yeast, the MS2 coat protein binds to the MS2 hairpin region of the hybrid RNA, thereby localizing the RNA to the promoter region. If the RBP of interest specifically recognizes the tested RNA sequence, it brings the transcriptional activation domain to the promoter, initiating transcription of the reporter gene (e.g. *lacZ* or *HIS3*) [115]. In addition to validating known RNA–protein interactions, such as those between IRP1 (Iron Regulatory Protein 1) and the HIV Tat protein with their cognate RNA substrates, the Y3H system has also been used to discover novel RNA–protein interactions, by screening either protein or RNA libraries against known RNAs or RBPs, respectively [116]. For example, Y3H has been employed to identify binding sequences of Snp1 (homolog of the human U1-70K protein) [117], SLBP (Stem Loop Binding Protein) [118], FBF [119], and Mpt5p (member of PUF family) [120]. Additionally, it was applied to engineer Pumilio proteins with novel binding specificities and to detect interaction between long non-coding RNA (lncRNA) *DANA2* with transcription factor ERF84, which regulates plant drought response [121–123]. The Y3H screening has also been optimized to enable screening of many-by-many protein–RNA interactions [124–126]. One of the limitations of the Y3H system is that the tested RNA can sometimes activate transcription on its own, without binding to a protein partner carrying a transactivation domain. In fact, this property has been exploited to identify RNA-based transcriptional activators [127,128]. Despite limitations such as frequent false positives (due to non-specific protein–protein, RNA–protein, or DNA–protein interactions), reliance on the expression of multiple components, and the localization of RNA in a non-native context (which can alter molecular environment and partner recruitment), the Y3H platform remains a versatile and scalable tool for studying RNA–protein interactions *in vivo*. The limited transformation capacity of the Y3H system can be addressed by combining it with SELEX, whereby initial selection is carried out *in vitro* to reduce the sequence space subjected to *in vivo* screening [129]. Recently, the conceptually similar Bacterial Three-Hybrid (B3H) system has been developed, expanding the application of the Y3H approach to other species. Given the high transformation efficiency of *E. Coli* (10^9^-10^10^ versus 10^5^-10^6^ CFU/μg of DNA in yeast), this system can be a powerful tool for screening large protein and RNA libraries [130–132]. Additionally, the recently developed CRISPR-Hybrid system uses CRISPR to recruit RNA to genomic loci in bacteria and has been applied to discover novel RNA aptamers recognizing RBPs [133].

### Immunoprecipitation-based MPBAs (MPRNA-RIP and MPRNA-IP)

Massively Parallel RNA assay followed by RNA immunoprecipitation (MPRNA-RIP) was developed to identify RNA elements that specifically interact with splicing factor hnRNPK in mammalian cells [134]. In this approach, a library of RNA fragments (~110–140 nucleotides) is inserted into the 3′UTR of a synthetic GFP mRNA and transfected into mammalian cells. After cell lysis, the RBP is immunoprecipitated using specific antibodies, and the enriched RNA fragments associated with the protein are identified by sequencing [134] (Figure 2(C)). Recently, this approach has been applied to explore the binding requirements of Pumilio 1 and Pumilio 2, as well as to measure their effect on the translational efficiency of reporters [135]. Additionally, a conceptually similar strategy, called MPRNA-IP, has been developed in which RNA tiles are inserted directly downstream of the CMV promoter and therefore, do not encode any reporter protein. In MPRNA-IP, tested sequences of 157 nucleotides are systematically mutagenized enabling comprehensive exploration of both sequence and structural requirements for RBP binding. In contrast to MPRNA-RIP, which is performed under native conditions without crosslinking, MPRNA-IP employs formaldehyde crosslinking to stabilize RNA–protein interactions. Using MPRNA-IP, binding preferences were characterized for Pumilio 2-PRE (Pumilio response elements), MCP-MS2, and TERT-hTR (human telomerase RNA) interactions. In summary, MPRNA-RIP and MPRNA-IP are efficient approaches that repurpose traditional MPRAs to assay RBP binding. However, both require IP-grade antibodies, and the quality of the results depends on the efficiency of immunoprecipitation as well as the stability of RNA–protein complexes during purification (for MPRNA-RIP), and can have biases introduced by formaldehyde crosslinking (for MPRNA-IP).

### RBPscan

Recently, RBPscan, a novel MPBA based on RNA editing to measure RNA–protein interactions *in vivo*, was developed [136]. This method builds upon TRIBE (Targeted RNA Identification By Editing), in which an RBP of interest is fused to the catalytic domain of ADAR (adenosine deaminase acting on RNA; ADAR_CD_), an enzyme that converts adenosines (A) to inosines (I) through deamination [137]. When an RBP-ADAR_CD_ fusion is expressed in cells, the RBP directs ADAR to its mRNA targets, which are then modified and identified via sequencing by detecting A-to-G conversions, as inosine chemically resembles guanine (Figure 2(D)). In contrast to TRIBE, RBPscan uses a synthetic mRNA library encoding reporter proteins (e.g. EYFP, Enhanced Yellow Fluorescent Protein), with a variable sequence region placed in the 3′UTR, instead of relying on endogenous mRNAs. Critically, the mRNA reporter contains a region with multiple adenosines positioned adjacent to the variable RNA element, serving as a sensor for RBP binding [138]. This design overcomes key limitations of TRIBE, such as its reliance on sparse endogenous adenosines in an appropriate context for editing, which often results in false negatives. Moreover, by using a library of mRNAs with a customized variable region, RBPscan allows for the systematic exploration of a large sequence space. Reporter mRNAs are localized in the cytoplasm and resemble the natural structure of endogenous mRNAs, including the 5′UTR, CDS, 3′UTR, and poly(A) tail, which is conducive to mimicking natural RNA–protein interactions. The variable region and adenosine-rich regions are flanked by constant sequences containing Illumina adapters, allowing straightforward preparation of NGS libraries. RBPscan has been successfully used for *de novo* motif discovery for several RBPs, both from a fully randomized library containing 7–8 variable positions (~10^4^ variants) and from a systematically mutagenized library of PREs [136]. The latter, combined with tightly controlled expression of Pumilio-ADAR_CD_ fusion protein, enabled the generation of *in vivo* binding curves and the quantification of relative *K*_D_ values. Importantly, RBPscan possesses a high dynamic range enabled by multiple editing positions and allows detection of binding affinities from the nanomolar to millimolar range. The system has been successfully demonstrated in zebrafish embryos, mammalian cells, and yeast. Furthermore, inclusion of a reporter gene within the mRNA allows simultaneous quantification of RBP binding and its effect on mRNA translation, thereby linking *cis*- and *trans*-regulatory elements to their functional outcomes within a single assay. Recently, a similar approach was described that uses an engineered adenosine deaminase (TadA) to profile both the RNA and protein components of the boxB–λN system [139]. The use of different RNA editors with distinct substrate preferences may enable multiplexed RBP profiling in the future. Despite these advances, RNA-editing-based approaches require careful implementation of controls to ensure reproducibility and minimize false-positive editing events. These controls include monitoring expression levels of all components and incorporating an ADAR_CD_–only control or a catalytically inactive ADAR variant to assess background editing.

## Challenges and future directions

With the advancement of DNA synthesis and sequencing technologies, tools to analyse RNA–protein interactions at scale have become increasingly accessible. Among these, *in vitro*-based MPBAs have dominated the field. Their key advantage lies in the ability to examine the binding of extremely large numbers of sequence variants under precisely controlled biochemical conditions. While these methods initially focused on identifying only the top binding motifs, they have since evolved to enable the profiling of RBP binding landscapes across vast sequence spaces and the measurement of absolute kinetic parameters. Nevertheless, in most *in vitro* approaches a single RBP is typically tested, with only a few methods allowing interrogation of two or more proteins [21,63,76]. In cells, however, many RBPs function as part of multiprotein complexes, by which they can influence each other’s ability to recognize specific RNA motifs and affect the dynamics of interaction [40,140–143]. Additionally, most *in vitro* MPBAs test only isolated fragments of RBPs, as purifying full-length proteins is often challenging, particularly because they frequently contain IDRs [20]. However, growing evidence suggests that IDRs can contribute directly to RNA binding, mediate interactions with partner proteins, and serve as targets for regulatory mechanisms [18–20,144]. Moreover, *in vitro* binding dynamics are often primarily governed by the dissociation rate (*K*_OFF_), as there is little to no competition from other factors. The cellular environment, on the other hand, is markedly more complex, shaped by molecular crowding, competing interactions, post-translational modifications, and cooperative binding [17,37,40,63]. Under these conditions, the association of RBPs with their RNA targets can become the limiting step in complex formation. Therefore, while *in vitro* MPBAs provide valuable insights into RNA–protein dynamics, we argue that their results must ultimately be tested and validated *in vivo* using full-length proteins.

Despite their importance, MPBAs capable of profiling RBP binding specificities and kinetics *in vivo* remain in their infancy. Progress in this area has been limited primarily by the complexity of the cellular environment, which makes it difficult to interrogate specific RNA–protein interactions independently from the influence of other factors present in the cell. Additionally, delivering large libraries of sequence variants into cells remains technically demanding, and the number of variants that can be profiled *in vivo* is several orders of magnitude lower than *in vitro*. The Y3H system represents the widely used *in vivo* method developed to date for this purpose. However, it also suffers from significant limitations, including a high rate of false positives and the artificial localization of tested RNAs to promoter regions, potentially distorting the natural context of RNA–protein interactions. In this regard, approaches such as MPRNA-RIP and RBPscan, which enable the testing of RBP-RNA interactions in 3′UTRs, are promising as they may better mimic the natural molecular environment. However, one must consider the inherent complexity of the intracellular milieu, in which diverse RNAs, RBPs, and co-factors form intricate interaction networks. This complexity can complicate the interpretation of binding events and hinder the ability to distinguish direct RNA–protein interactions from indirect or co-factor-assisted binding.

Additionally, *in vivo* MPBAs have been largely overshadowed by CLIP and RNA-editing based approaches. While these methods are highly informative for identifying endogenous RNA targets of RBPs, they are not always well suited for deciphering the precise binding motifs of RBPs. Moreover, with the exception of one example [44], they do not provide the quantitative data necessary to assess the affinity or kinetic landscape of RBPs. Therefore, we see the need to develop novel MPBAs, that employ innovative strategies to bring quantitative approaches into the *in vivo* context. We also anticipate that developing tools for precise control and quantification of RBP expression in cells will help bring these approaches closer to biochemical-level precision. Additionally, combining complementary methods provides a powerful approach for the detailed characterization of RBPRNA interactions. In this framework, *in vitro* approaches can serve as a starting point, where a broad pool of sequences is progressively narrowed down and subsequently tested *in vivo*. The strategy of combining two independent methods to achieve more comprehensive RBP characterization has been applied previously, for example, by fusing SELEX with Y3H, or by integrating Bind-n-Seq with eCLIP (enhanced CLIP) in the context of the ENCODE project [84,129].

Traditional MPRAs have been widely used to dissect the contribution of regulatory RNA *cis*-elements across various cellular contexts, revealing their roles in RNA stability, localization, and translational efficiency [26–29,31,32]. However, the development of MPRA-based approaches to systematically identify and quantify the binding and functional activity of *trans*-acting factors on their corresponding *cis*-elements has lagged. The availability of both *in vivo* and *in vitro* techniques to identify RBP binding motifs has greatly enhanced our ability to profile binding specificities and predict interactions with identified *cis*-elements. However, such approaches are often insufficient. MPRAs and MPBAs are frequently performed in cellular contexts that differ from those in which the regulatory activity is ultimately interrogated, or even *in vitro*, making it difficult to establish a direct causal link between *cis-* and *trans-* elements. Bridging this gap is essential for understanding the dynamic interplay between RNA sequences and the proteins or complexes that recognize them. This can be achieved using methods such as RBPscan, which incorporate built-in sensors to monitor RBP binding and reporter protein expression simultaneously.

RBPs are increasingly recognized as key players in various diseases, often harbouring mutations that affect their function. Although CLIP-based approaches are now being scaled up to study many RBPs in parallel [145], they remain limited in their ability to analyse multiple variants of the same RBP because most available antibodies cannot distinguish proteins that differ by only a few amino acids. MPBAs, with their capacity to explore large protein and RNA sequence spaces, hold great promise in this regard. However, most current MPBA methods are optimized for exploring RNA sequence variation rather than systematically analysing protein variants. This underscores the need for new approaches that can introduce and evaluate mutations within RBPs to better understand how these alterations affect RNA binding specificity and affinity.

Finally, given the vast amount and diversity of data generated by MPBAs, it is essential to discuss standards for data annotation and deposition. We advocate for minimal reporting standards for MPBAs to improve reproducibility and comparability between different approaches. At a minimum, datasets should specify: (i) the design and sequence composition of the library, (ii) detailed information about the RBP construct, including sequence boundaries, tags, and any post-translational modifications, and (iii) the concentrations of all components and the experimental time points. Authors should deposit both the raw sequencing data and all processed outputs, including ranked motif tables, derived binding models, and the exact code and parameters used for motif inference or kinetic modeling. Standardized reporting will allow results to be more readily reused by other researchers and enable comparisons across different experimental platforms. Ultimately, such datasets could be integrated into resources such as oRNAment, which can currently predict RBP binding sites transcriptome-wide based on RNAcompete and RBNS data [146]. Furthermore, standardized data annotation and deposition would facilitate the development of robust computational models capable of accurately interpreting MPBA datasets and predicting RNA–protein interactions that have not yet been experimentally tested. Large language models are poised to play an instrumental role in integrating and analysing this information, as demonstrated by their successful application to RNAcompete datasets [78], and will further empower machine learning approaches that combine MPBA-derived motif and functional data to develop predictive models of RBP binding and regulatory activity.

## Concluding remarks

Over the years, the toolkit for studying RNA–protein interactions using MPBAs has become increasingly diverse, enabling the profiling of these interactions both *in vitro* and *in vivo*. We anticipate that MPBAs will continue to evolve and play an instrumental role in deciphering the mechanisms of post-transcriptional regulation. MPBAs hold unique power for understanding the detailed principles that govern RNA–protein interactions. They can provide crucial insights relevant for structural biology studies, advance protein engineering for biotechnological and therapeutic applications, and also support *in vivo* observations obtained with other methods (e.g. CLIP or RNA-editing-based approaches) by shedding light on the fine mechanistic details underlying these interactions. With many techniques now available, the choice of method depends on the specific biological question, the available technical infrastructure, and cost considerations.

## Abbreviations

*cis*-elements: DNA or RNA sequence elements located within the same molecule they regulate (e.g. splicing enhancers, localization signals, miRNA binding sites).
*trans*-elements: Typically proteins or RNAs that interact with DNA or RNA molecules to regulate their function (e.g. TFs, RBPs, miRNAs).
MPBAs: *Massively Parallel Binding Assays*: High-throughput techniques that assess RNA-protein interactions using large libraries of RNA or protein variants.
MPRAs: *Massively Parallel Reporter Assays*: Approaches that measure the functional output (e.g. expression, stability) of RNA regulatory elements in a high-throughput manner.
*k*-mer: A nucleotide sequence of length *k*; commonly used to describe motifs or substrings in RNA or DNA sequences.
Binding equilibrium: The state at which the rates of association and dissociation between an RBP and RNA are equal, allowing determination of binding affinity (e.g. dissociation constant, *K*_D_).
Sequence space: The total set of all possible sequence variants that can be explored or sampled in an experiment.
PWMs: *Position Weight Matrices*: A matrix-based representation of nucleotide frequency or binding preference at each position in a motif, often used to model RBP binding sites.

## Data Availability

Data sharing is not applicable to this article as no new data were created or analysed in this study.

## Outstanding questions

- How can we effectively identify complex or bipartite RNA motifs, especially those that are discontinuous or context-dependent?
- How do RNA–protein interaction kinetics differ between *in vitro* and *in vivo* contexts?
- How can *in vivo* systems be adapted to accurately quantify dissociation constants (*K*_D_)?
- How does RNA structure influence RBP binding specificity and affinity?
- How do multiple RBDs within a single RBP cooperate to recognize extended or structured RNA elements?
- How can we analyse the composition of RNP complexes and their effects on RBP binding and function?
